# Inflammation, sauna bathing, and all-cause mortality in middle-aged and older Finnish men: a cohort study

**DOI:** 10.1007/s10654-022-00926-w

**Published:** 2022-10-18

**Authors:** Setor K. Kunutsor, Sae Young Jae, Sudhir Kurl, Jussi Kauhanen, Jari A. Laukkanen

**Affiliations:** 1 National Institute for Health Research Bristol Biomedical Research Centre, University Hospitals Bristol and Weston NHS Foundation Trust and the University of Bristol, Bristol, UK; 2 Musculoskeletal Research Unit, Translational Health Sciences, Bristol Medical School, University of Bristol, Learning & Research Building (Level 1), Southmead Hospital, Bristol, BS10 5NB UK; 3 Diabetes Research Centre, Leicester General Hospital, University of Leicester, Gwendolen Road, Leicester, LE5 4WP UK; 4 Graduate School of Urban Public Health, University of Seoul, Seoul, Republic of Korea; 5 Department of Sport Science, University of Seoul, Seoul, South Korea; 6 Department of Urban Big Data Convergence, University of Seoul, Seoul, Republic of Korea; 7 Institute of Public Health and Clinical Nutrition, University of Eastern Finland, Kuopio, Finland; 8 Department of Medicine, Institute of Clinical Medicine, University of Eastern Finland, Kuopio, Finland; 9 Department of Medicine, Central Finland Health Care District, Hospital District, Jyvaskyla, Finland

**Keywords:** Sauna, Inflammation, C-reactive protein, Mortality, Cohort study

## Abstract

Inflammation and sauna bathing are each related to the risk of all-cause mortality. The interplay between inflammation, sauna bathing and all-cause mortality is not well understood. We aimed to evaluate the separate and joint associations of inflammation (high sensitivity C-reactive protein, hsCRP) and frequency of sauna bathing (FSB) with all-cause mortality in a cohort of Caucasian men. We used the Kuopio Ischaemic Heart Disease Study cohort comprising 2575 men aged 42–61 years at baseline. Serum hsCRP was measured using an immunometric assay and sauna bathing habits were assessed by a self-administered questionnaire. High sensitivity CRP was categorized as normal and high (≤ 3 and > 3 mg/L, respectively) and FSB as low and high (defined as ≤ 2 and 3–7 sessions/week respectively). A total of 1618 deaths occurred during a median follow-up of 27.8 years. Comparing high vs normal hsCRP levels, the multivariable-adjusted HR (95% CI) for all-cause mortality was 1.27 (1.13–1.44). Comparing high vs low FSB, the multivariable-adjusted HR (95% CI) for all-cause mortality was 0.86 (0.76–0.97). Compared with normal hsCRP-low FSB, high hsCRP-low FSB was associated with an increased risk of all-cause mortality 1.28 (1.12–1.47), with no evidence of an association for high hsCRP-high FSB and all-cause mortality risk 1.06 (0.81–1.40). Positive additive and multiplicative interactions were found between hsCRP and FSB in relation to mortality. In a general Finnish male population, both hsCRP and FSB are each independently associated with all-cause mortality. However, frequent sauna baths appear to offset the increased all-cause mortality risk related to high hsCRP levels.

## Introduction

Mortality is the oldest, simplest and most widely used summary measure of population health. Many factors contribute to mortality. In the Global Burden of Disease Study 2019, among 20 top risk factors, the leading five risks for attributable global deaths were high systolic blood pressure, tobacco use, dietary risks, high fasting plasma glucose, and air pollution [1]. Though several epidemiological observational cohort studies have demonstrated significant associations between individual risk markers and overall mortality, fewer studies have assessed the joint contributions of these risk markers on this endpoint.

It is well known that inflammatory processes are involved in the pathogenesis of several acute and chronic conditions as well as vascular and nonvascular diseases. Hence, the consistent associations demonstrated between elevated levels of circulating inflammatory markers such as C-reactive protein (CRP) and interleukin-6 (IL-6) and the risk of cause-specific and all-cause mortality is not unexpected [2, 3] Sauna bathing, a passive heat therapy and an activity that has been a tradition in Finland for thousands of years, has mainly been used for the purposes of pleasure and relaxation [4]. The health benefits of frequent sauna bathing are becoming well documented. Accumulating epidemiological and intervention studies suggest that Finnish sauna bathing may be protective of several adverse nonfatal vascular and non-vascular outcomes [5–10]. Higher frequency and duration of sauna bathing have also been shown to be strongly, inversely, and independently associated with fatal cardiovascular as well as all-cause mortality events [4, 11–16]. In addition to potentiating the beneficial effects of protective factors such as physical fitness (high cardiorespiratory fitness, CRF levels) [11, 13, 17], emerging evidence suggests that high frequency of sauna bathing (FSB) may offset the adverse effects of other risk factors. For instance, our group has previously shown that high FSB can offset the increased risk of pneumonia due to inflammation or low socioeconomic status (SES) [18, 19]. We hypothesize that there exists an interplay between sauna bathing, inflammation, and overall mortality. Whether frequent sauna baths could attenuate or offset the increased risk of mortality due to inflammation has not yet been previously investigated. In this context, using a population-based prospective cohort of 2575 middle-aged to older Finnish men, we aimed to (1) evaluate the joint effects of inflammation (as measured by high-sensitivity CRP, hsCRP) and FSB on the risk of all-cause mortality and (2) confirm the existing associations of hsCRP and FSB with all-cause mortality.

## Materials and methods

Participants utilized for this analysis were part of the Kuopio Ischemic Heart Disease (KIHD) cohort, an ongoing population-based prospective study comprising a representative sample of men aged 42–61 year recruited from Kuopio or its surrounding rural communities in eastern Finland. The study design, recruitment and assessment methods have been described in previous reports [11, 12]. Briefly, 2682 eligible men agreed to participate in the study and underwent screening and baseline examinations between March 1984 and December 1989. In the current analysis, we excluded men with missing data on exposures and covariates (n = 107), leaving 2575 men with complete data on hsCRP, sauna bathing habits, covariates and all-cause mortality outcomes. The Research Ethics Committee of the University of Eastern Finland approved the study protocol and written informed consent was obtained from all participants. The investigation was conducted in accordance with the principles outlined in the Declaration of Helsinki and its future amendments.

For measurements of blood-based biomarkers, blood samples were collected in the morning between 8 and 10 a.m. after participants fasted overnight and abstained from alcohol consumption for at least 3 days and from smoking for at least 12 h. Measurement of the cholesterol content of lipoprotein fractions employed enzymatic methods (Boehringer Mannheim, Germany). Serum hsCRP was measured using an immunometric assay (Immulite High-Sensitivity CRP assay, DPC) [20]. Self-administered questionnaires were used to assess sociodemographic and lifestyle characteristics such as smoking, alcohol consumption and socioeconomic status (SES), existing medical conditions and medication history [21]. The weekly frequency of sauna bathing sessions was assessed based on a traditional Finnish sauna which has air with a relative humidity of 10–20% [11–13]. The assessment of SES involved the generation of a summary score comprising important indicators such as income, education, occupational prestige, material standard of living and housing conditions [22–24]. The items for each indicator were scored and summed. The composite SES score ranged from 0 to 25, with higher values indicating lower SES. Alcohol consumption was assessed using the Nordic Alcohol Consumption Inventory and was reported in g/week. Prevalent coronary heart disease (CHD) was defined as a history of previous myocardial infarction, angina pectoris, the use of nitroglycerin for chest pain once a week or more frequently or chest pain. Prevalent type 2 diabetes (T2D) was defined as a fasting blood glucose level ≥ 7.0 mmol/L or clinical diagnosis of diabetes with dietary, oral, or insulin treatment. We included all deaths that occurred from study entry through to 2018. Mortality events were ascertained by data linkage to the Finnish National Death Registry utilizing the Finnish social security number that is used by all registries.

Using descriptive statistics, baseline characteristics were reported as means (standard deviation, SD) or medians (interquartile range, IQR) for continuous variables and counts (percentages) for categorical variables. Cox proportional hazards models were used to estimate multivariable-adjusted hazard ratios (HRs) with 95% CIs for all-cause mortality following confirmation of no major departure from the proportionality of hazards assumptions using Schoenfeld residuals [25]. Adjustment for covariates was based on three progressive models: (Model 1) age; (Model 2) Model 1 plus body mass index (BMI), systolic blood pressure (SBP), total cholesterol, high-density lipoprotein cholesterol (HDL-C), smoking status, history of T2D, history of CHD, alcohol consumption, SES, and physical activity; and (Model 3) Model 2 plus mutual adjustment for each exposure. These covariates were selected based on their previously established roles as traditional risk factors for mortality, previous research evidence including the KIHD study [11, 26, 27], or their potential as confounders based on known associations with mortality and observed associations with the exposures [28]. To maintain consistency with previous reports [11, 13, 19], hsCRP was categorized as normal and high (≤ 3 and > 3 mg/L, respectively) and FSB as low and high (defined as ≤ 2 and 3–7 sauna sessions per week respectively). For the evaluation of the joint associations, study participants were divided into four groups according to categories of hsCRP and FSB: normal hsCRP-low FSB; normal hsCRP-high FSB; high hsCRP-low FSB; and high hsCRP-high FSB. Interactions between hsCRP and FSB were examined on both the additive and multiplicative scales in relation to all-cause mortality risk, as discussed in previous reports [29, 30]. Additive interactions were assessed using the “relative excess risk due to interaction” (RERI), computed for binary variables as RERI_HR_ = HR_11_ − HR_10_ − HR_01_ + 1 [31], where HR_11_ is the HR of the outcome (i.e., all-cause mortality) if both risk factors (high hsCRP and low FSB) are present, HR_10_ is the HR of the outcome if one risk factor is present and the other is absent (high hsCRP and high FSB), with HR_01_ being vice versa (normal hsCRP and low FSB). Multiplicative interactions were assessed using the ratio of HRs = HR_11_/(HR_10_ × HR_01_) [31]. A positive additive interaction is indicated if RERI > 0 and a positive multiplicative interaction is indicated if the ratio of HRs > 1. All statistical analyses were conducted using Stata version MP 17 (Stata Corp, College Station, Texas) and statistical significance was set at *p* < 0.05.

## Results

**Fig. 1:**
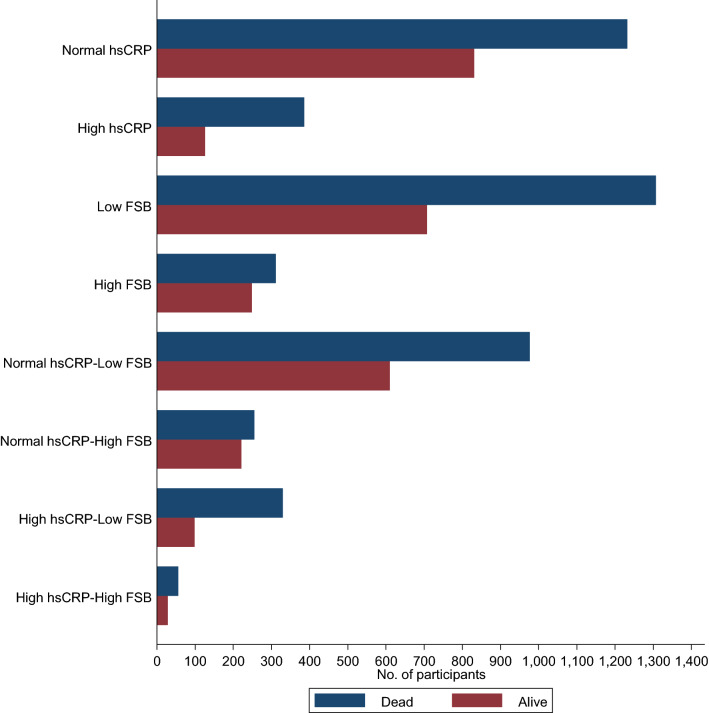
Men who were dead or alive at the end of follow-up by exposure categories. *FSB* frequency of sauna bathing; *hsCRP* high sensitivity C-reactive protein

The overall mean (SD) age of men at baseline was 52 (5) year. The median (IQR) of hsCRP was 1.29 (0.71–2.48) mg/L. The frequency of sauna use ranged from 0–7 sessions/week, with a median (IQR) of 2 (1–2) sessions/week (Table 1). Frequency of sauna bathing was weakly and inversely correlated with hsCRP (r = −0.06; *p* < 0.001). Only 12 men did not use sauna at all. During a median (IQR) follow-up of 27.8 (18.4–31.1) yr, 1618 deaths occurred. Cardiovascular causes constituted 744 (46.0%) of the total deaths. Figure 1 shows the number of men who died or were alive in each exposure category at the end of follow-up.

**Table 1:** Baseline characteristics of study participants (N = 2575 men)

Characteristics	Mean (SD) or median (IQR) or n (%)
High sensitivity C-reactive protein (mg/l)	1.29 (0.71–2.48)
Frequency of sauna bathing (sessions/week)	2 (1–2)
*Questionnaire/prevalent conditions*	
Age (year)	53 (5)
Alcohol consumption (g/week)	31.8 (6.3–91.5)
Socioeconomic status	8.49 (4.23)
Current smoking	814 (31.6)
History of type 2 diabetes	104 (4.0)
History of coronary heart disease	649 (25.2)
*Physical measurements*	
BMI (kg/m^2^)	26.9 (3.6)
SBP (mmHg)	134 (17)
DBP (mmHg)	89 (11)
Physical activity (KJ/day)	1204 (628–2000)
*Blood biomarkers*	
Total cholesterol (mmol/l)	5.91 (1.08)
HDL-C (mmol/l)	1.29 (0.30)
Fasting plasma glucose (mmol/l)	5.36 (1.27)

*BMI* body mass index, *CHD* coronary heart disease, *DBP* diastolic blood pressure, *HDL-C* high-density lipoprotein cholesterol, *IQR* interquartile range, *SD* standard deviation, *SBP* systolic blood pressure

**Fig. 2:**
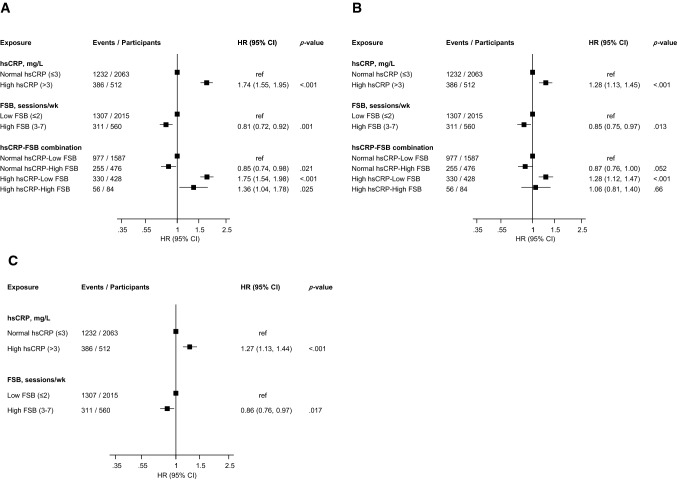
Separate and joint associations of high sensitivity C-reactive protein and frequency of sauna bathing with risk of all-cause mortality in men. *CI* confidence interval; *FSB* frequency of sauna bathing; *HR* hazard ratio; *hsCRP* high sensitivity C-reactive protein; *ref* reference. **A** Adjusted for age. **B** Model 1 plus body mass index, systolic blood pressure, total cholesterol, high-density lipoprotein cholesterol, smoking status, history of type 2 diabetes, history of coronary heart disease, alcohol consumption, socioeconomic status, and physical activity. **C** Model 2 plus FSB for hsCRP or hsCRP for FSB

Compared with men with normal hsCRP, high hsCRP was associated with an increased risk of all-cause mortality following adjustment for age 1.74 (95% CI 1.55–1.95) (Fig. 2A), which was attenuated to 1.28 (95% CI 1.13–1.45) on further adjustment for BMI, SBP, total cholesterol, HDL-C, smoking status, history of T2D, history of CHD, alcohol consumption, SES, and physical activity (Fig. 2B). The association persisted on further adjustment for FSB 1.27 (95% CI 1.13–1.44) (Fig. 2C). On adjustment for the covariates in Fig. 2B, high FSB was associated with a decreased risk of all-cause mortality compared with low FSB 0.85 (95% CI 0.75–0.97) (Fig. 2B), which remained similar on additional adjustment for hsCRP 0.86 (95% CI 0.76–0.97) (Fig. 2C).

Compared with normal hsCRP-low FSB, multivariable analysis showed that high hsCRP-low FSB was associated with an increased risk of all-cause mortality 1.28 (95% CI 1.12–1.47), with no evidence of an association for high hsCRP-high FSB and all-cause mortality risk 1.06 (95% CI 0.81–1.40) (Fig. 2B). Results of interaction analysis showed the RERI was 0.1 and the ratio of HRs was 1.05, indicating the presence of additive and multiplicative interactions.

## Comment

In this general population-based study of middle-aged to older Finnish men, high levels of hsCRP and high FSB were independently associated with an increased and decreased risk of all-cause mortality, respectively. These findings were consistent with previous reports [2, 11]. These associations persisted on mutual adjustment for each exposure. New findings from the KIHD cohort study based on the joint associations of hsCRP and FSB showed that the risk of all-cause mortality was increased in men with elevated hsCRP and low FSB, but the increased risk of all-cause mortality due to elevated hsCRP was attenuated by high FSB. Interaction analysis showed the association between the combined exposures (i.e., combination of high hsCRP and low FSB) and all-cause mortality risk exceeded the sum or product of their associations considered separately.

Inflammation plays a major role in the development of several acute events and chronic disease states. C-reactive protein, a non-specific marker for inflammation which is produced by the liver, is known to be elevated in various inflammatory states. Though the evidence for the association between elevated CRP levels and increased all-cause mortality risk is robust and strong, there is no evidence of a causal relationship [32]. Rather, elevated CRP has been described as a marker of hidden, potentially serious chronic inflammatory disease [32]. It has been reported that the associations between CRP and all-cause mortality may reflect underlying chronic inflammatory conditions [32]. Furthermore, increased levels of biomarkers of inflammation such as CRP may reflect a final common biochemical pathway of poor health status, which subsequently leads to death from cardiovascular and non-cardiovascular causes in old age [33]. There is evidence suggesting that the adaptations produced by an ordinary sauna bath corresponds to that produced by moderate or high intensity physical activity [34, 35]. The mechanistic pathways postulated to underlie the association between frequent passive heat exposure (eg, regular sauna bathing) and decreased risk of death include reduction in blood pressure [36]; improvement in endothelial function [37, 38]; reduction in oxidative stress [39, 40]; beneficial modulation of the autonomic nervous system [41] and in levels of circulating cardiovascular risk factors such as natriuretic peptides [42]; improved arterial stiffness; decreased intima media thickness [39, 42–44]; and substantial improvement in cardiovascular function [45–47].

These findings add to the emerging evidence on the ability of frequent sauna baths to promote longevity and attenuate or offset the adverse effects of cardiovascular risk factors [18, 19]. Passive whole body heat therapies such as Finnish sauna bathing are now emerging as relaxation activities that may have therapeutic potential for preventing and treating adverse health outcomes. Given that sauna bathing is more commonly available in Nordic countries, it is not known whether potential health implications may be applicable in other populations. However, there is increasing research on the potential health benefits of sauna baths on a global scale and it is emerging as a common activity in many other countries [48, 49]. Further research is warranted into the mechanistic pathways underlying the beneficial effects of sauna exposure on adverse health outcomes including mortality and how this can be translated into clinical practice.

This is the first evaluation of the interplay between inflammation, sauna bathing and all-cause mortality. Other strengths include the population-based prospective cohort design, the large sample and long-term follow-up, and adjustment for a comprehensive panel of potential confounders. The limitations included the inability to generalzse the results to other populations and the potential for observational design biases such as residual confounding, reverse causation and regression dilution bias. Though the findings were based on only men, it is likely that the results will be similar in women given similar effects of inflammation and sauna bathing on adverse cardiovascular outcomes including mortality in women in previous studies [12, 16, 50].

In conclusion, both hsCRP and FSB are each independently associated with all-cause mortality risk in a general Finnish male population. There is also an interplay between inflammation, sauna bathing, and all-cause mortality –the current study shows interactive effects of inflammation and sauna bathing on the risk of mortality and frequent sauna baths appear to offset the increased all-cause mortality risk related to high hsCRP levels.

## Data Availability

The data used for this study are available from the corresponding author upon reasonable request.
